# A single center report of MScanFit motor unit number estimation in five muscles of healthy subjects

**DOI:** 10.3389/fnhum.2022.1078848

**Published:** 2023-01-17

**Authors:** Xiaohui Song, Lijun Cui, Ya Zong, Maoqi Chen, Zhiyuan Lu, Qing Xie, Ping Zhou

**Affiliations:** ^1^Department of Rehabilitation, Ruijin Hospital, Shanghai Jiao Tong University School of Medicine, Shanghai, China; ^2^School of Rehabilitation Science and Engineering, University of Health and Rehabilitation Sciences, Qingdao, Shandong, China

**Keywords:** motor unit number estimation (MUNE), MScanFit, single center, compound muscle action potential (CMAP), CMAP scan

## Abstract

The objective of this study was to estimate the number of motor units in 5 muscles from healthy individuals using the MScanFit program based on compound muscle action potential (CMAP) scan recordings. The examined muscles included first dorsal interosseous (FDI), abductor pollicis brevis (APB), abductor digiti minimi (ADM), second lumbrical (SL), and abductor hallucis (AH). CMAP scans were recorded from a total of 24 healthy participants. Motor unit number estimation (MUNE) values were derived from the MScanFit program. The average MUNE was 136.1 ± 31.1 (mean ± standard deviation) for the FDI, 134.9 ± 37.4 for the APB, 127.3 ± 32.3 for the ADM, 39.6 ± 8.3 for the SL, and 143.9 ± 28.9 for the AH muscles. Findings of the study provide useful information of the MScanFit MUNE for the examined muscles of healthy subjects from a single center.

## 1. Introduction

MScanFit is a motor unit number estimation (MUNE) method based on compound muscle action potential (CMAP) scan and a model simulation of the responses (Bostock, 2016; Jacobsen et al., 2018a). Since its introduction, the method has been used to estimate motor unit number in both upper and lower limb muscles. Most of the previous studies have focused on examining or tracking motor unit loss in amyotrophic lateral sclerosis and other neuromuscular diseases or disorders [see a brief review (Tankisi, 2021)]. In this study we set to investigate MScanFit MUNE in five muscles of healthy subjects in a single center in China and assess their consistence with other reports in literature.

## 2. Methods

### 2.1. Subjects

Neurologically intact subjects were recruited for this study through word of mouth or recruitment posters placed at affiliated Ruijin Hospital of Shanghai Jiao Tong University School of Medicine (Shanghai, China). The inclusion criteria were: age between 21 and 50 years, without known history of peripheral nerve or muscular disorders. A total of 24 subjects (13 males, mean age 29.5 ± 6.9 years, and 11 females, mean age 29.5 ± 5.7 years) were recruited. The mean age of all subjects was 29.5 ± 6.2 years (range: 21–43 years). Twenty-two subjects were right-handed and two were left-handed. All subjects participated in the APB study; 23 subjects participated in the FDI, ADM, SL and AH studies. Standard electrodiagnostic conduction studies for each muscle confirmed that the latency, amplitude, and conduction velocity were in the normal range for all the subjects. The study protocol was approved by the Institutional Review Board of Shanghai Jiao Tong University. All participants gave written informed consent before the experiment.

### 2.2. Experiment

Five muscles were examined including the first dorsal interosseous (FDI) muscle, the abductor pollicis brevis (APB) muscle, the abductor digiti minimi (ADM) muscle, the second lumbrical (SL) muscle, and the abductor hallucis (AH) muscle. For each muscle, the subject's dominant side was examined. Skin temperature was maintained above 32°C. Subjects were asked to remain completely relaxed during the recording. Alcohol pads were used to clean the skin before placing the electrodes. The stimulating electrode was a standard bar surface electrode that has two contact surfaces 20 mm apart, with each of them 9 mm in diameter. For each muscle, the optimal stimulus site was determined shifting the electrode position, so a large M wave or compound muscle action potential (CMAP) can be recorded at a relatively low stimulus intensity. Once this position was determined, the electrode was secured in place with surgical tape and self-adherent wrap. The cathode of the electrode was positioned distally. The active electrode and the reference electrode were disposable Ag–AgCl surface electrodes, which were 13 mm in diameter with an extended portion for connecting to the amplifier with an alligator clip. The electrodes were coated with conductive paste before placed. A self-adhesive electrode was used as the ground. The electrode placement for CMAP scan recording was detailed below for each of the examined muscle. All data were collected using a Nicolet EDX EMG system (Natus Neurology Incorporated, Middleton, WI, USA).

**Figure 1:**
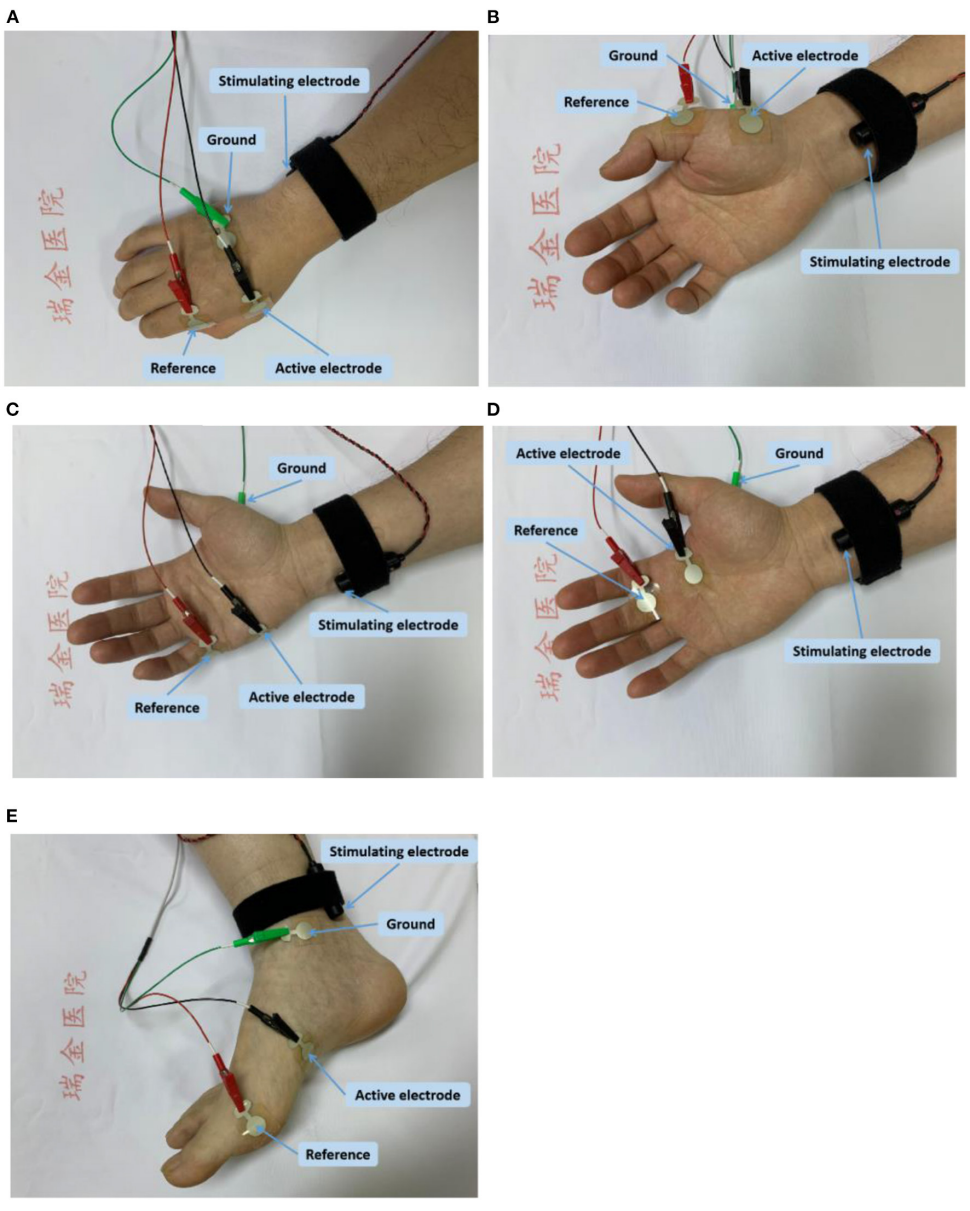
Electrode placement for CMAP scan of each examined muscle; **(A)** the FDI muscle; **(B)** the APB muscle; **(C)** the ADM muscle; **(D)** the SL muscle; and **(E)** the AH muscle.

FDI: The active electrode was placed on the FDI muscle and the reference electrode was placed on the distal phalanx of thumb. The ground electrode was placed on the dorsal side of the hand. The stimulating electrode was placed 1–2 cm proximal to the wrist, for delivering electrical stimuli to ulnar nerve (Figure 1A).

APB: The active electrode was placed on the APB muscle, and the reference electrode was placed on the metacarpal phalangeal joint of the thumb. The ground electrode was placed on the dorsum of the hand. The stimulating electrode was placed 1–2 cm proximal to the wrist for activating median nerve (Figure 1B).

ADM: The active electrode was placed on the ADM muscle, and the reference electrode was placed on the metacarpal phalangeal joint of the little finger. The ground electrode was placed on the dorsum of the hand. The stimulating electrode was placed 1–2 cm proximal to the wrist, for delivering electrical stimuli to ulnar nerve (Figure 1C).

SL: The active electrode was placed on the second lumbrical muscle, and the reference electrode was placed on the surface of the proximal interphalangeal joint of the middle finger. The ground electrode was placed on the palm of hand. The stimulating electrode was placed on above the transverse carpal ligament (between the flexor carpi radialis tendon and the palmaris longus tendon) for delivering electrical stimuli to median nerve (Figure 1D).

AH: The active recording electrode was positioned approximately 1 cm below and proximal to the navicular tubercle, and the reference electrodes was positioned on the metatarsophalangeal joint of the big toe. The ground electrode was placed at the medial malleolus. The stimulating electrode was placed posterior to the medial malleolus to stimulate tibial nerve (Figure 1E).

A nerve conduction study for each of the examined muscles was first performed. Then the CMAP scan program equipped with the EMG machine was used to record the progressive recruitment of all motor units from repetitive stimulations. An automatic search of the electrical stimulation range was performed to determine S0 and S100 in order to cover the entire recruitment range. Then the CMAP scan started using a protocol of 0.1 ms stimulus pulse duration, 500 steps, 2 Hz stimulus frequency, and a linear decline mode for the stimulus intensity.

### 2.3. Data analysis

The free MScanFit program developed by Bostock was used for estimating motor unit number based on each CMAP scan data (Bostock, 2016; Jacobsen et al., 2018a). The default setting of the program was used. The MUNE values with percentage error < 7% of the fitting were accepted.

## 3. Results

**Figure 2:**
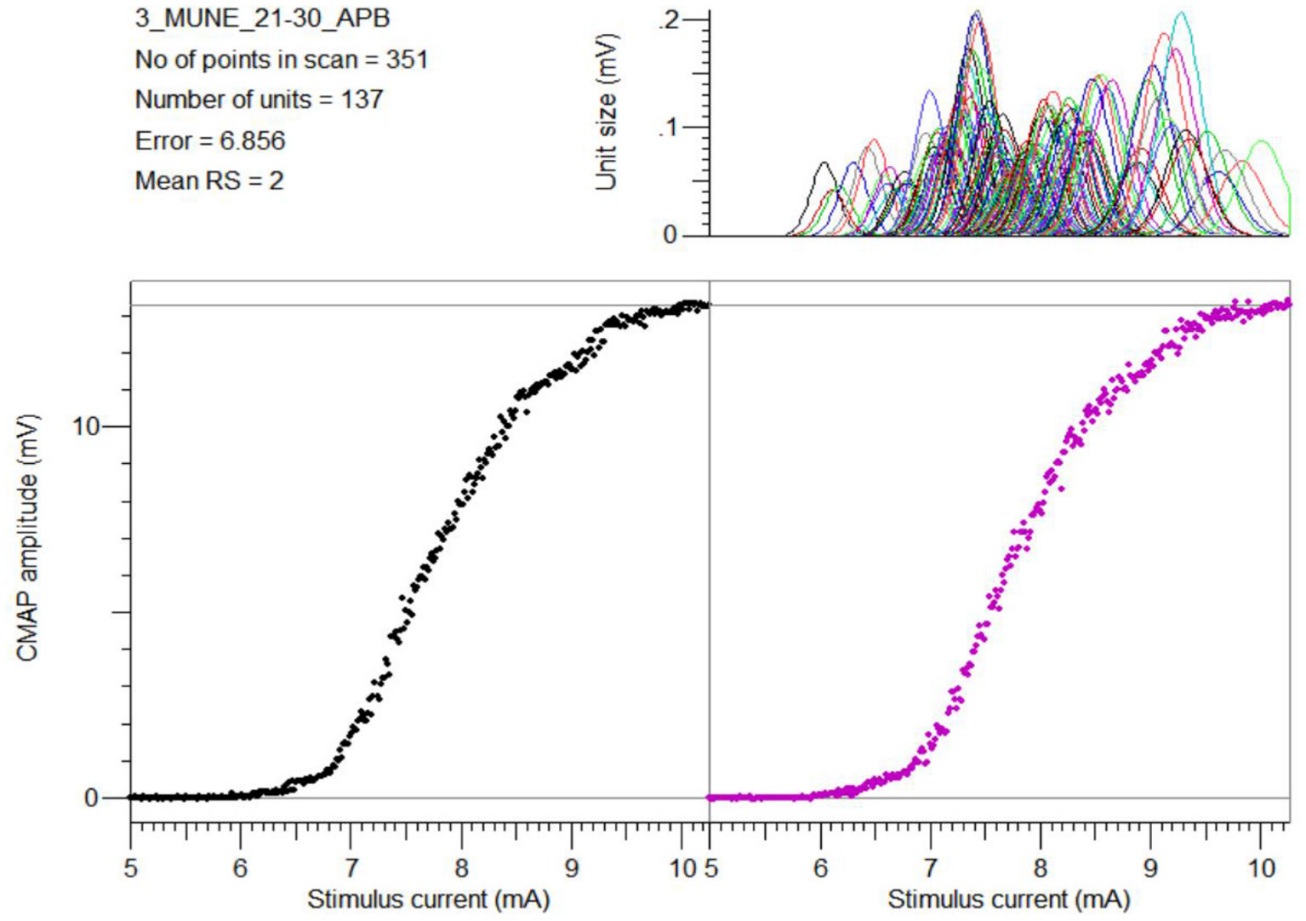
An example of the experimental CMAP scan from the APB muscle **(bottom left)**, the modeled CMAP scan using the MScanFit program **(bottom right)**, and the estimated motor unit action potential distribution **(top right)**.

All subjects tolerated the CMAP scan procedures well. Table 1 summarizes the parameters derived from CMAP scans for each of the examined muscles. Figure 2 shows a typical example of the experimental and modeled CMAP scans from one of the examined muscles.

**Table 1:** CMAP scan analysis of 5 muscles (mean ± standard deviation).

	**FDI**	**APB**	**ADM**	**SL**	**AH**
S0 (mA)	6.9 ± 1.6	6.1 ± 2.9	5.9 ± 1.7	7.5 ± 3.3	11.1 ± 3.8
S100 (mA)	17.7 ± 4.3	15.4 ± 4.9	16.0 ± 5.1	18.3 ± 4.4	35.3 ± 8.4
CMAP (mV)	15.3 ± 2.6	11.3 ± 2.5	11.1 ± 2.0	1.9 ± 0.5	20.6 ± 4.7
MUNE	136.1 ± 31.1	134.9 ± 37.4	127.3 ± 32.3	39.6 ± 8.3	143.9 ± 28.9
Largest unit (μV)	489.2 ± 256.5	439.2 ± 209.6	347.3 ± 116.4	101.4 ± 21.6	552.9 ± 258.6
Mean unit (μV)	118.1 ± 37.7	90.6 ± 25.5	88.6 ± 24.2	43.7 ± 5.1	146.4 ± 52.3

CMAP, compound muscle action potential; MUNE, motor unit number estimation; FDI, first dorsal interosseous; APB, abductor pollicis brevis; ADM, abductor digiti minimi; SL, second lumbrical; and AH, abductor hallucis.

## 4. Discussion

This study reports reference values of MScanFit MUNE in five muscles from a single center in China. MScanFit has also been used by other centers to estimate the motor unit number in these muscles from healthy control subjects. For the FDI muscle, Higashihara et al. (2020) reported similar CMAP amplitude but moderately lower MUNE, which might be due to wider stimulus pulse width (0.2 ms) compared with the current study (0.1 ms). Gunes et al. (2021) reported a larger extent of lower CMAP amplitude and MUNE, which can be in part attributed to relatively old subject ages (55.8 ± 12.3 years) in their study.

Perhaps the APB has been the most often studied muscle using CMAP scan. The average MScanFit MUNE of the APB muscle in healthy subjects was mostly reported in a range between 90 and 120 (Farschtschi et al., 2017; Jacobsen et al., 2017, 2018b; Kristensen et al., 2019; Sirin et al., 2019; Bennedsgaard et al., 2020; Higashihara et al., 2020; Schneider et al., 2021; Sørensen et al., 2022). Our results are higher than most previous results. The mean CMAP amplitude in our study was also slightly higher than most previous studies. The ADM is also a frequently examined muscle using CMAP scan. The previously reported MScanFit MUNE of the ADM muscle in healthy subjects had a wide span from 60 to 131 (Sirin et al., 2019; Kesim-Sahin et al., 2020; Gunes et al., 2021; Sørensen et al., 2022). Our results appeared to be at the high end of this range, although the CMAP amplitude for the ADM muscle was in between previous studies.

Compared with the APB and ADM muscles, there are rather limited MUNE studies of the AH and SL muscles. Our MUNE results on AH were similar to those found by Witt et al., while the CMAP amplitude was slightly smaller (Witt et al., 2020). Compared with another MScanFit MUNE study on AH by Li et al. (2018), our results were similar in CMAP amplitude but slightly higher in MUNE.

The results on MScanFit MUNE of the SL muscle were similar to a previous study in a United States (US) center also performed by our group, although the CMAP amplitude was slightly smaller in the current study (Zong et al., 2022a). To the best of our knowledge, these are only two studies on MUNE of the SL muscle. The relatively low motor unit number of the SL muscle can facilitate single motor unit extraction, providing a favorable feature for motor unit investigations.

The difference in MScanFit MUNE between our research center and other centers can be due to a variety of factors such as experimental protocols for CMAP scans (e.g., stimulus pulse duration) (Maathuis et al., 2012; Zong et al., 2020, 2022b; Sleutjes et al., 2021), recording conditions, MScanFit program parameter setting, subject age difference, variability for individual subjects, and experimenter factors, etc. In fact, we also performed MScanFit studies in a US center for both APB and FDI muscles. An unexpected finding was that the MScanFit MUNE performed in the US research center was smaller for both FDI and APB muscles, while the CMAP amplitude recorded at the US center was higher for both FDI and APB muscles (Zong et al., 2021, 2022b), although the same experimental protocols (stimulus pulse duration, steps, etc.) and MScanFit program parameter setting were used. In addition, the subject ethnicity, age, and experimenter factors can be excluded to have a significant effect. We speculate this difference might be partly related to different electrode size, individual subject difference between the two centers, or other relevant factors that need further investigation. Limitations of the current study include a lack of test-retest reliability analysis and a relatively small subject number for investigating reference values of each muscle.

## Data availability statement

The raw data supporting the conclusions of this article will be made available by the authors, without undue reservation.

## Ethics statement

The studies involving human participants were reviewed and approved by the Institutional Review Board of Shanghai Jiao Tong University. The patients/participants provided their written informed consent to participate in this study.

## Author contributions

Study design: QX, YZ, and PZ. Data collection: XS, LC, and YZ. Data analysis and interpretation: YZ, MC, ZL, XS, LC, QX, and PZ. Writing—original draft preparation: XS and LC. Writing—revision: PZ. Writing—review and editing: YZ, MC, ZL, and QX. Study supervision: QX and PZ. All authors have read and agreed to the published version of the manuscript.
